# The efficacy and safety of methylprednisolone in hepatitis B virus-related acute-on-chronic liver failure: a prospective multi-center clinical trial

**DOI:** 10.1186/s12916-020-01814-4

**Published:** 2020-12-08

**Authors:** Lin Jia, Ran Xue, Yueke Zhu, Juan Zhao, Juan Li, Wei-Ping He, Xiao-Mei Wang, Zhong-Hui Duan, Mei-Xin Ren, Hai-Xia Liu, Hui-Chun Xing, Qing-Hua Meng

**Affiliations:** 1 Department of Critical Care Medicine of Liver Disease, Beijing You-An Hospital, Capital Medical University, Beijing, China; 2 Key laboratory of Carcinogenesis and Translational Research (Ministry of Education/Beijing), Department of phase I clinical trial, Peking University Cancer Hospital & Institute, Fucheng Road 52, Haidian District Beijing, 100142 China; 3 302 Hospital of People’s Liberation Army, Liver Disease Center for Military Staff, Beijing, China; 4 Institute of Infectious Diseases, Beijing Di Tan Hospital, Capital Medical University, No. 8 Jing Shun Dong Street, Chao yang District, Beijing, 100015 China

**Keywords:** Hepatitis B, Acute-on-chronic liver failure, Methylprednisolone

## Abstract

**Background:**

Hepatitis B virus-related acute-on-chronic liver failure (HBV-ACLF) is a severe condition with high mortality due to lack of efficient therapy. Until now, the use of methylprednisolone (MP) in HBV-ACLF is still controversial. We aimed to evaluate the efficacy and safety of MP in HBV-ACLF.

**Methods:**

Totally 171 HBV-ACLF patients from three medical centers were randomly allocated into MP group (83 patients treated with MP intravenously guttae for 7 days plus standard treatment: 1.5 mg/kg/day [day 1–3], 1 mg/kg/day [day 4–5], and 0.5 mg/kg/day [day 6–7]) and control group (88 patients treated with standard treatment). The primary endpoints were 6-month mortality and prognostic factors for 6-month survival. The survival time, cause of death, adverse events, liver function, and HBV DNA replication were analyzed.

**Results:**

The 6-month mortality was significantly lower in MP group than control group [32.4% vs. 42.5%, *P* = 0.0037]. MP treatment was an independent prognostic factor for 6-month survival [HR (95% CI) 0.547(0.308–0.973); *P* = 0.040]. Factors associated with reduced 6-month mortality in MP group included HBV DNA and lymphocyte/monocyte ratio (LMR) (*P* < 0.05). Based on ROC curve, LMR+MELD had a better predictive value for prognosis of HBV-ACLF under MP treatment. No significant difference in HBV DNA replication was observed between groups (*P* > 0.05).

**Conclusions:**

MP therapy is an effective and safe clinical strategy in HBV-ACLF, increasing the 6-month survival rate.

Clinical trials registered at http://www.chictr.org.cn as ChiCTR-TRC-13003113 registered on 16 March 2013.

**Supplementary information:**

**Supplementary information** accompanies this paper at 10.1186/s12916-020-01814-4.

## Background

In Asia, hepatitis B virus-related acute-on-chronic liver failure (HBV-ACLF) accounts for about 70% of all ACLF cases, which is identified with severe acute exacerbation (AE) of liver function to liver failure in the chronic hepatitis B (CHB) patients [1, 2], with high mortality (51.6 to 54.3%) [3]. Liver transplantation remains the only curative treatment for ACLF with limited application [4–6]. Up to now, there is no effective treatment which has been developed for HBV-ACLF patients.

Overwhelming systemic inflammation and susceptibility to infection are two key features of ACLF [7, 8]. “Endotoxin-macrophage-cytokine storm” is the core pathogenesis of liver failure. The chemical essence of endotoxin is lipopolysaccharide (LPS) [9]. With the interaction of LPS-binding protein, it binds to a variety of cell membrane receptor, stimulating the synthesis and release of cytokines, involving interferon-α (INF-α) and IL-12. As the most commonly used immunosuppressive and anti-inflammatory agent, methylprednisolone (MP) has theoretical basis for the treatment of ACLF [10–12]. However, until now, the use of methylprednisolone (MP) in HBV-ACLF is still uncertain and controversial [13].

With the coming of nucleoside analogs (NAs), more and more guidelines have recommended NAs to be used in patients with acute exacerbation of chronic HBV infection. The early combined use of NAs and MP could be a good option to reverse the potential deterioration in patients with HBV-related liver failure. Our previous study has demonstrated that MP can improve 28-day survival rate in HBV-ACLF patients [14]. A recent study has also reported that early combination therapy with glucocorticoids (GCs) and NAs induces rapid resolution of inflammation in ALF due to transient HBV infection [15]. However, Huang C et al. [16] investigated retrospectively the efficacy of GCs in patients with HBV-ACLF, which indicated that GCs treatment did not improve transplant-free survival in patients with HBV-ACLF.

Therefore, in order to further evaluate the efficacy and safety of MP in HBV-ACLF patients, we proceed this multi-center, prospective randomized controlled clinical trial to provide evidence for MP as one of the clinically effective treatments for HBV-ACLF.

## Methods

### Eligibility

**Fig. 1:**
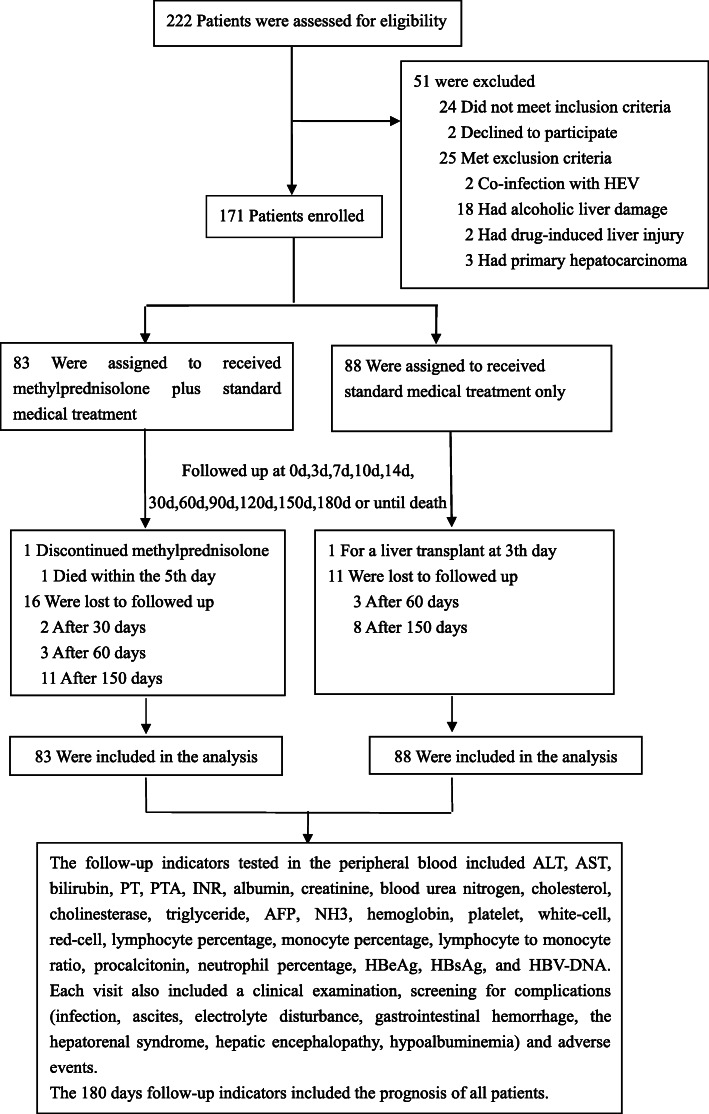
Flowchart for the enrollment of patients with HBV-ACLF

Patients were recruited at Beijing Di Tan Hospital, Capital Medical University, People’s Liberation Army No. 302 Hospital, and Beijing You-An Hospital, Capital Medical University, from April 2013 to May 2015. All procedures related to this research were accorded morally with current laws as well as the creeds of the Declaration of Helsinki. The research was permitted by the Ethical Committee of Beijing You-An Hospital, Capital Medical University, Beijing Di Tan Hospital Capital Medical University, and People’s Liberation Army No. 302 Hospital (No.2 [2013]). All study participants gave their informed consent to participate in the study. The process of study selection and exclusion was shown in Fig. 1.

### Patients selection

The inclusion criteria were the following: (1) aged 18 years or older; (2) previously diagnosed or undiagnosed HBV, HBsAg positive; and (3) all enrolled patients met the criteria for ACLF from the consensus recommendations of the Asian Pacific Association for the Study of the Liver (APASL) specified as follows: an acute hepatic insult manifesting as jaundice (serum bilirubin ≥ 5 mg/dl [≥ 85 μmol/l]) and coagulopathy (INR ≥ 1.5 or prothrombin activity ≤ 40%) complicated within 4 weeks by clinical ascites and/or encephalopathy in a patient with previously diagnosed or undiagnosed chronic liver disease/cirrhosis [17, 18]. Diagnostic criteria for cirrhosis are made by history, physical examination, and previously available laboratory, fibrosis biomarkers (e.g., FIB-4 or FibroTest), endoscopic or radiologic investigations (ultrasound, CT abdomen or transient elastography [fibroscan]), or a previously liver biopsy history [19].

The exclusion criteria comprised the following: (1) uncontrolled bacterial infection or gastrointestinal hemorrhage before enrollment; (2) infection with hepatitis virus other than HBV, or human immunodeficiency virus; (3) autoimmune diseases, alcoholic liver disease, and drug-induced hepatitis; and (4) serious renal, cardiac, respiratory, neurologic diseases, or any detectable tumor.

The diagnostic criteria of complications included (1) gastrointestinal hemorrhage confirmed by endoscopy, (2) bacterial infection diagnosed by a positive culture result [20], (3) fungal infection diagnosed according to EORTC/MSG definition [21], (4) hepatorenal syndrome (HRS) diagnosed according to the International Ascites Club’s guidelines [22], (5) spontaneous bacterial peritonitis (SBP) diagnosed based on diagnostic paracentesis [23], (6) electrolyte disturbance defined as ≥ 1 electrolyte abnormalities of K+, Na+, and Cl−, (7) hypoalbuminemia diagnosed when albumin < 35 g/L, and (8) pleural effusion diagnosed by X rays or computerized tomography.

### Study design

After investigators confirmed eligibility, patients were randomized (1:1 allocation ratio) to MP plus standard management (MP group) or standard treatments (control group) by computer-generated permutated block randomization (block size of four) stratified. Treatment was started when patients were enrolled and the day of enrollment was defined as day 1.

Patients were followed up at the baseline (0 days), 3 days, 7 days, 10 days, 14 days, 30 days, and then monthly until the 6th month. Collected information included laboratory tests, screening for complications. The alive status means significant improvement in clinical symptoms, bilirubin< 5 × upper limit of normal (5ULN), PTA > 30%, or INR < 1.5.

### Treatment protocols

Standard treatments included antiviral drugs (lamivudine 100 mg/day, adefovir dipivoxil 10 mg/day, entecavir 0.5 mg/day, telbivudine 600 mg/day, or tenofovir disoproxil fumarate 300 mg/day based on individual’s condition before enrollment), nutritional support (1.5–2.0 g protein/kg/day and 35–40 kcal/kg/day), plasma exchange, and complications control such as anti-infection, administration of human serum albumin (10 g per day until serum albumin was 35 g/L), fresh frozen plasma (200 ml to 400 ml/day until the INR < 1.5), and vasoactive agents (terlipressin alone or in combination with noradrenaline to reverse septic shock). All treatments were performed based on the criteria of diagnostic and treatment guidelines for ACLF adopted by the Chinese Medical Association [24].

The MP group was given standard treatments combined with MP intravenously guttae for 7 days: 1.5 mg/kg/day, days 1–3; thereafter 1 mg/kg/day, days 4–5; and followed by 0.5 mg/kg/day, days 6–7.

### Clinical and laboratory parameters

Laboratory tests measured PTA, prothrombin time (PT), INR, bilirubin, aspartate aminotransferase (AST), alanine aminotransferase (ALT), albumin, creatinine, blood urea nitrogen, cholesterol, cholinesterase, triglyceride, alpha-fatal protein (AFP), blood ammonia, hemoglobin, platelet, white blood cell, red blood cell, lymphocyte percentage, monocyte percentage, lymphocyte to monocyte ratio (LMR), and procalcitonin and neutrophil percentage with routine automated techniques. HBsAg and HBeAg were assayed using commercially available radioimmunoassay kits (Roche Diagnostics). Serum HBV-DNA was quantified using a cross linking chemical hybridization assay (Roche Diagnostics) and the detection threshold is 100 copies/ml. All hospitals use the same assay and standard operating procedure for the above indicators. Model for end-stage liver disease (MELD) score was calculated according to the original formula proposed by the Mayo Clinic [25].

### Study outcomes

The primary endpoints were 6-month mortality and prognostic factors for 6-month survival. The secondary endpoints were adverse events and changes of laboratory indices during treatment.

### Statistical analysis

Quantitative variables, expressed as means ± SD or medians (interquartile range), were compared using Wilcoxon, Kruskal–Wallis, or Student’s *t* tests, as appropriate. Qualitative variables, expressed as percentages, were compared using chi-square or Fisher’s exact tests. Ordinal enumeration variables were analyzed by rank sum tests. The changes of indices during follow up time were compared using variance analysis for repeated data. Kaplan-Meier survival curves were plotted and compared with log-rank tests. Significantly predictive factors of mortality in univariate models (*P* < 0.1) were included in multivariate Cox-regression models. ROC curve was used to identify the predictive value of indices for prognosis. All analyses were performed using SPSS (version 26.0, Chicago, IL, USA), and statistical significance was set up as a two-sided *P* value < 0.05. The formula was used to calculate the sample size: n1 = n2 = $\frac{\left({q}_1^{-1}+{q}_1^{-1}\right){\left({Z}_{\alpha /2}+{Z}_{\beta}\right)}^2{S}^2}{\delta^2}$, where (*α* = 0.05, *β* = 0.10, 1-β = 0.90, *σ* = 8.8, *S* = 15.9). The required sample size was “70” for each group. Improvement of PTA by at least 30% is proposed to make a difference on the curative effect [26].

## Results

### Baseline characteristics of patients with HBV-ACLF

The process of study selection and exclusion was shown in Fig. 1. A total of 222 patients were evaluated. After exclusion of 51 patients who did not meet the inclusion criteria, 171 cases were enrolled. Among 171 patients who were randomized (mean age, 45.2 years; 152 (88.9%) men), 142 (83.0%) completed follow-up through 6 months. Table 1 shows the baseline demographic and clinical characteristics for both groups. Most patients did not apply NAs treatment when the disease onset (69.9% in the MP group and 78.4% in the control group). Twenty-one patients (12.3%) had undetectable HBV DNA (< 100 copies/ml) at the baseline. There was no significant difference between MP group and control group at the baseline characteristics (Table 1).

**Table 1:** Baseline characteristics and complications of patients

Characteristic	No. of patients (*N* = 171)	Methylprednisolone (*N* = 83)	Control (*N* = 88)	*P*
Male^a^	152 (88.9%)	74 (8.4%)	78 (88.6%)	0.914
Age (years)	45.2 ± 12.3	43.7 ± 12.7	46.6 ± 11.8	0.121
Cirrhosis^a^	139 (81.3%)	67 (80.7%)	72 (81.8%)	0.854
Time from onset^d^ to admission (days)^b^	20 (10–30)	16 (10–21)	20 (13–30)	0.083
MELD score	23.3 ± 4.7	22.8 ± 4.0	23.9 ± 5.2	0.132
Lg HBV DNA (IU/ml)	4.8 ± 1.9	4.9 ± 2.0	4.6 ± 1.8	0.383
HBV DNA^a^				
Positive	150 (87.7%)	70 (84.3%)	80 (90.9%)	0.191
Negative	21 (12.3%)	13 (15.7%)	8 (9.1%)	0.191
HBeAg^a^				
Positive	90 (52.6%)	43 (51.8%)	47 (53.4%)	0.834
Negative	81 (47.4%)	40 (48.2%)	41 (46.6%)	0.834
HBsAg^b^	2582.0 (401.0–6012.0)	2582.0 (422.8–5884.0)	2613.0 (325.4–6197.3)	0.843
No medication with NAs when the disease onset^a^	127 (74.3%)	58 (69.9%)	69 (78.4%)	0.202
Never used before	89 (52.0%)	38 (45.8%)	51 (58.0%)	
Drug withdrawal	38 (22.2%)	20 (24.1%)	18 (20.5%)	
ADV	6 (3.5%)	2 (2.4%)	4 (4.5%)	
ETV	16 (9.4%)	12 (14.5%)	4 (4.5%)	
LAM	10 (5.8%)	4 (4.8%)	6 (6.8%)	
ETV+ADV	3 (1.8%)	1 (1.2%)	2 (2.3%)	
LAM+ADV	3 (1.8%)	1 (1.2%)	2 (2.3%)	
Medication with NAs when the disease onset^a^	44 (25.7%)	25 (30.1%)	19 (21.6%)	0.202
LAM	7 (4.1%)	4 (4.8%)	3 (3.4%)	
LAM+TDF	1 (0.6%)	1 (1.2%)	0	
LAM+ADV	7 (4.1%)	3 (3.6%)	4 (4.5%)	
ETV	26 (15.2%)	16 (19.3%)	10 (11.4%)	
ADV	2 (1.2%)	0	2 (2.3%)	
LdT	1 (0.6%)	1 (1.2%)	0	
NAs therapy after enrollment^a^	171 (100%)	83 (100%)	88 (100%)	0.101
LAM	16 (9.4%)	6 (7.2%)	10 (11.4%)	
LAM+ADV	7 (4.1%)	5 (6.0%)	2 (2.3%)	
LAM+TDF	1 (0.6%)	1 (1.2%)	0	
ETV	142 (83.0%)	69 (83.1%)	73 (83.0%)	
ETV+ADV	2 (1.2%)	0	2 (2.3%)	
ADV	1 (0.6%)	0	1 (1.1%)	
LdT	2 (1.2%)	2 (2.4%)	0	
ALT (U/L)^b^	307.0 (117.2–573.6)	347.0 (131.7–685.9)	276.3 (106.8–514.1)	0.080
AST (U/L)^b^	214.0 (156.8–485.0)	291.3 (156.8–515.0)	184.4 (155.5–306.1)	0.054
Bilirubin (μmol/L)	353.9 ± 138.4	335.3 ± 133.8	371.4 ± 141.2	0.089
Albumin (g/L)	31.0 ± 4.1	31.4 ± 3.6	30.7 ± 4.5	0.257
Cholinesterase (IU/L)	3215.8 ± 1165.5	3262.0 ± 1063.3	3172.3 ± 1258.9	0.616
AFP (ng/ml)^b^	80.0 (33.1–180.0)	86.0 (45.5–240.0)	65.7 (21.7–132.8)	0.150
Cholesterol (mmol/L)	2.3 ± 0.8	2.4 ± 0.9	2.2 ± 0.7	0.059
Blood urea nitrogen (mmol/L) ^b^	4.0 (3.2–6.1)	4.6 (3.1–6.1)	4.3 (3.5–6.3)	0.054
Creatinine (μmol/L)^b^	66.2 (56.8–81.0)	64.4 (55.4–76.6)	68.0 (58.1–84.5)	0.123
NH3 (μg/dl)	86.5 ± 45.2	89.2 ± 44.9	84.0 ± 45.7	0.449
Triglyceride (mmol/L)^b^	0.9 (0.6–1.5)	0.8 (0.5–1.7)	0.9 (0.6–1.4)	0.936
White blood cell (× 10^9^/L)	7.3 ± 3.5	7.3 ± 3.4	7.3 ± 3.6	0.960
Lymphocyte percentage	20.7 ± 8.5	20.1 ± 8.1	21.3 ± 8.9	0.372
Monocyte percentage	9.5 ± 3.6	9.0 ± 3.5	9.8 ± 3.7	0.147
Lymphocyte/monocyte ratio^b^	2.2 (1.5–3.0)	2.2 (1.5–3.0)	2.1 (1.5–2.9)	0.502
Neutrophil percentage	68.6 ± 10.6	69.1 ± 9.6	68.1 ± .11.4	0.547
Procalcitonin (μg/L)^b^	0.6 (0.4–0.9)	0.6 (0.5–1.0)	0.6 (0.3–0.8)	0.197
PT (s)^b^	22.9 (20.2–27.9)	22.9 (19.2–22.8)	22.9 (20.7–27.6)	0.877
PTA (%)	35.9 ± 9.2	36.0 ± .10.3	35.8 ± 8.1	0.865
INR^a^	2.9 (1.7–2.3)	2.1 (1.6–2.6)	2.0 (1.7–2.2)	0.580
Red blood cell (× 10^12^/L)	3.9 ± 0.8	4.0 ± 0.8	3.8 ± 0.9	0.070
Hemoglobin (g/L)	124.2 ± 21.1	127.6 ± 18.9	121.5 ± 22.3	0.056
Platelet (×10^9^/L)	105.2 ± 51.6	113.0 ± 45.3	97.9 ± 56.1	0.055
Ascites^a^	103 (60.2%)	45 (54.2%)	58 (65.9%)	0.118
Infections^a^	92 (53.8%)	47 (56.6%)	45 (51.1%)	0.472
Electrolyte disturbance^a^	17 (9.9%)	5 (6.0%)	12 (13.6%)	0.096
Encephalopathy^a^	21 (12.3%)	12 (14.5%)	9 (10.2%)	0.400
Hypoalbuminemia^a^	35 (20.5%)	16 (19.3%)	19 (21.6%)	0.708
Hepatorenal syndrome^a^	5 (2.9%)	1 (1.2%)	4 (4.5%)	0.369
Pleural effusion^a^	7 (4.1%)	3 (3.6%)	4 (4.5%)	1.000
Hyperthyroidism^a^	1 (0.6%)	1 (1.2%)	0	0.485
Peptic ulcer^a^	2 (1.2%)	1 (1.2%)	1 (1.1%)	1.000
Artificial liver support after enrollment^c^	–	87.98	84.13	0.582
Artificial centesis after enrollment^c^	–	79.55	92.08	0.090
The time of antibiotic use after enrollment^c^	–	91.72	80.60	0.141

^a^Data expressed with number of cases

^b^Data expressed as median (range)

^c^Date expressed with mean rank

^d^Onset: appearance of nausea, poor appetite, jaundice, or gastrointestinal hemorrhage

### Standard treatments of HBV-ACLF

Standard treatments include antiviral drugs, plasma exchange, anti-infection drugs, and artificial centesis. No significant difference was observed between MP group and control group for standard treatments after enrollment (Table 1). 19.3% and 12.5% of patients received intensive care therapy in MP group and control group, respectively (*P* = 0.173). 50.6% (42/83) patients in MP group received plasma exchange and 47.7% (42/88) patients in control group. There was no significant difference for the use of plasma exchange between groups (*P* = 0.707).

### Six-month primary outcome analysis

#### Mortality and causes-of-death analysis

**Fig. 2:**
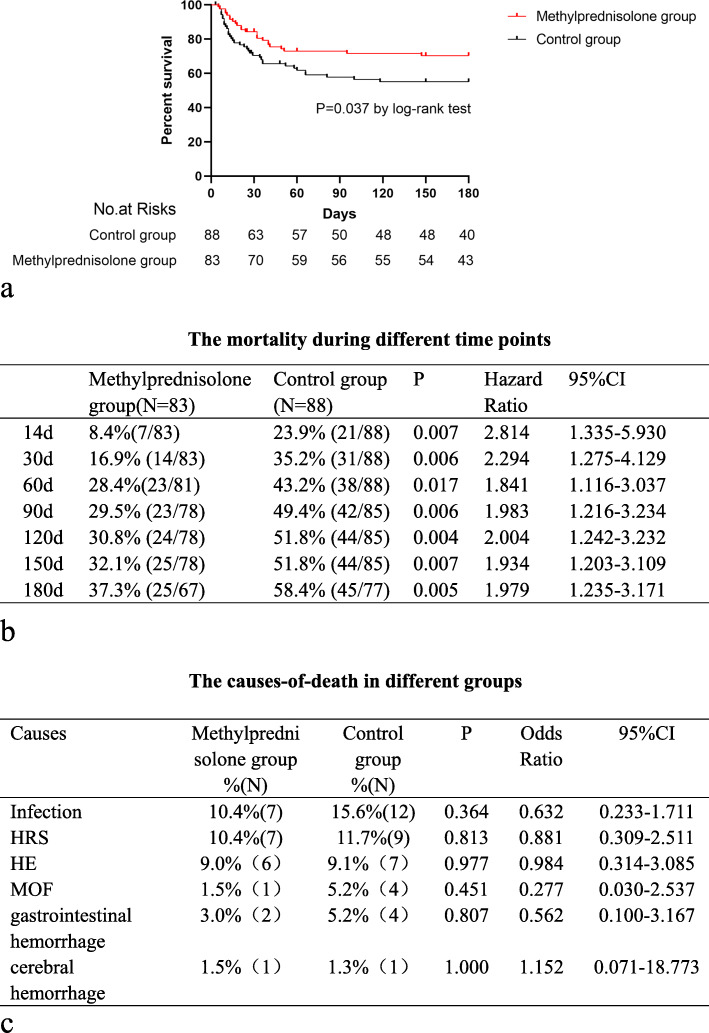
**a** A significant survival advantage was observed during 6 months among patients who received methylprednisolone. **b** The mortality during different time. **c** The causes-of-death in different groups

At the 6-month primary end point, 88 patients had died. 43.2% occurred before day 30, 19.3% between day 30 and day 60, 11.4% between day 60 and day 90, and 26.1% between day 90 and 6-months. In the primary analysis, the mortality rate of MP group and control group was 32.4% and 42.5%, respectively (*P* = 0.0037) at 6 months (Fig. 2a). The mortality rate of MP group was lower than control group on 14 days, 30 days, 60 days, 90 days, 120 days, 150 days, and 180 days.

Nineteen patients died from infections, 13 from HE, 16 from HRS, 6 from gastrointestinal bleeding, 5 from multiple organ failure, and 2 from cerebral hemorrhage. No significant differences in the causes of death were observed between MP group and control group (Fig. 2b, c).

Overall, 9 patients had been liver transplanted. Liver transplantation considered statistical evaluation to be an equivalent of alive in this study. 1.5% (1/83) patients in the MP group received liver transplantation and 10.4% (8/88) patients in the control group. There was no significant difference for the use of liver transplantation between groups [HR (95% CI) 0.131 (0.016–1.074); *P* = 0.064]. The intention-to-treat concept was applied in this study.

#### Efficacy of MP and risk factors associated with mortality at 6 months

Based on univariate analysis and collinearity diagnosis, MP, age, bilirubin, blood urea nitrogen, creatinine, triglyceride, lymphocyte percentage, monocyte percentage, LMR, PTA, INR, ascites, HE, and HRS were included for multivariate analysis. It was shown that MP was one of the independent predictors for mortality in HBV-ACLF patient [HR (95% CI) 0.547(0.308–0.973); *P* = 0.040] (Table 2). Meanwhile, Cox analysis revealed that HBV DNA and lymphocyte/monocyte ratio (LMR) can predict mortality of patients undergoing MP treatment (*P* < 0.05, Tables 3 and 4).

**Table 2:** Analysis of factors associated with mortality at 6 months

Factors	Univariate		Multivariate	
	HR(95%CI)	*P* value	HR(95%CI)	*P* value
**Methylprednisolone**	0.584 (0.349–0.976)	0.040	0.547 (0.308–0.973)	0.040
Centers ^a^	–	0.683		
Cirrhosis	1.114 (0.566–2.194)	0.755		
Antiviral treatment ^a^	–	0.410		
Male	0.594 (0.293–1.205)	0.149		
Age (years)	1.024 (1.004–1.045)	0.019	1.012 (0.987–1.037)	0.359
Time from onset to admission (days)	1.008 (0.993–1.024)	0.304		
MELD score	1.123 (1.072–1.176)	0.000		
lg HBV DNA (IU/ml)	0.973 (0.851–1.112)	0.686		
HBV DNA positive	0.617 (0.313–1.218)	0.164		
HBeAg positive	0.726 (0.439–1.201)	0.213		
HBsAg	1.000 (1.000–1.000)	0.248		
ALT(U/L)	0.999 (0.999–1.000)	0.092		
AST(U/L)	1.000 (0.999–1.001)	0.859		
**Bilirubin (μmol/L**)	1.004 (1.002–1.005)	0.000	1.003 (1.001–1.005)	0.004
Albumin(g/L)	0.985 (0.924–1.050)	0.648		
Cholinesterase (IU/L)	1.000 (1.000–1.000)	0.893		
AFP (ng/ml)	0.999 (0.997–1.001)	0.228		
**Blood urea nitrogen (mmol/L)**	1.149 (1.049–1.260)	0.003	1.195 (1.018–1.402)	0.029
Creatinine (μmol/L)	1.006 (1.001–1.011)	0.020	0.995 (0.984–1.005)	0.318
NH3(μg/dl)	1.002 (0.997–1.008)	0.413		
Triglyceride (mmol/L)	0.628 (0.401–0.986)	0.043	0.905 (0.524–1.561)	0.719
Cholesterol (mmol/L)	0.835 (0.585–1.190)	0.319		
White blood cell(× 10^9^/L)	1.057 (0.986–1.133)	0.115		
Lymphocyte percentage	0.934 (0.903–0.966)	0.000	0.950 (0.883–1.023)	0.175
**Monocyte percentage**	1.103 (1.023–1.190)	0.011	1.149 (1.011–1.307)	0.034
Lymphocyte to monocyte ratio	0.497 (0.366–0.674)	0.000	0.838 (0.468–1.502)	0.553
Neutrophil percentage	1.037 (1.012–1.062)	0.003		
Procalcitonin (μg/L)	0.993 (0.871–1.132)	0.913		
**PTA (%)**	0.942 (0.914–0.970)	0.000	0.931 (0.870–0.996)	0.038
INR	2.272 (1.482–3.493)	0.000	0.918 (0.320–2.629)	0.873
Red blood cell(× 10^12^/L)	0.959 (0.711–1.293)	0.782		
Hemoglobin(g/L)	0.998 (0.986–1.009)	0.702		
Platelet(× 10^9^/L)	0.996 (0.991–1.002)	0.163		
Ascites	2.021 (1.154–3.539)	0.014	0.879 (0.467–1.656)	0.690
Infections	1.381 (0.826–2.308)	0.218		
Electrolyte disturbance	0.599 (0.217–1.651)	0.322		
HE	2.161 (1.148–4.066)	0.017	1.270 (0.629–2.564)	0.505
Hypoalbuminemia	1.010 (0.537–1.900)	0.975		
HRS	4.296 (1.544–11.954)	0.005	1.013 (0.216–4.753)	0.987

^a^Risk estimate statistics cannot be computed. They are only computed for two groups of data

**Table 3:** Association between different factors and 6-month mortality in the methylprednisolone group

Factors	Univariate		Multivariate	
	HR (95% CI)	*P*	HR (95% CI)	*P*
Male	0.496 (0.169–1.451)	0.200		
Centers^a^	–	0.575		
Antiviral treatment^a^	–	0.681		
Age (years)	1.006 (0.975–1.039)	0.695		
cirrhosis	1.254 (0.429–3.670)	0.679		
Time from onset to admission (days)	1.021 (1.001–1.043)	0.044	1.021 (0.997–1.045)	0.085
MELD scores	1.144 (1.053–1.244)	0.001		
lg HBV DNA	0.812 (0.653–1.008)	0.060		
**HBV DNA positive**	0.247 (0.105–0.584)	0.001	4.875 (1.596–14.889)	0.005
HBeAg positive	1.130 (0.506–2.523)	0.765		
HBsAg	1.000 (1.000–1.000)	0.184		
ALT(U/L)	0.999 (0.999–1.000)	0.213		
AST(U/L)	1.000 (0.999–1.001)	0.669		
Bilirubin (μmol/L)	1.005 (1.002–1.008)	0.001	1.001 (0.998–1.004)	0.534
Cholinesterase (IU/L)	1.000 (0.999–1.000)	0.375		
AFP (ng/ml)	0.997 (0.994–1.001)	0.111		
Albumin (g/L)	0.978 (0.872–1.096)	0.700		
Blood urea nitrogen (mmol/L)	1.094 (0.941–1.271)	0.241		
Creatinine (μmol/L)	1.000 (0.987–1.013)	0.976		
NH3 (μmol/L)	1.005 (0.998–1.012)	0.152		
Triglyceride (mmol/L)	0.731 (0.394–1.356)	0.320		
Cholesterol (mmol/L)	0.896 (0.537–1.496)	0.675		
White cell (× 10^9^/L)	1.049 (0.942–1.168)	0.381		
Red cell (× 10^9^/L)	0.996 (0.611–1.622)	0.986		
Hemoglobin (g/L)	0.998 (0.978–1.019)	0.870		
Platelet (× 10^9^/L)	0.999 (0.990–1.008)	0.851		
Lymphocyte percentage	0.955 (0.904–1.008)	0.096		
Monocyte percentage	1.181 (1.049–1.329)	0.006	1.156 (0.954–1.401)	0.138
**Lymphocyte/monocyte ratio**	0.533 (0.339–0.839)	0.007	0.537 (0.291–0.992)	0.047
Procalcitonin (μg/L)	0.992 (0.732–1.344)	0.957		
Neutrophil percentage	1.018 (0.978–1.060)	0.375		
PTA (%)	0.929 (0.887–0.974)	0.002	0.901 (0.790–1.028)	0.122
INR	2.764 (1.401–5.451)	0.003	0.613 (0.074–5.075)	0.650
Ascites	2.467 (1.021–5.959)	0.045	1.143 (0.398–3.276)	0.804
Infections	1.344 (0.588–3.072)	0.484		
Electrolyte disturbance	0.045 (0.000–64.307)	0.403		
HE	3.257 (1.347–7.873)	0.009	2.746 (0.868–8.683)	0.085
Hypoalbuminemia	1.183 (0.404–3.464)	0.759		
HRS	6.965 (0.899–53.954)	0.063		

^a^Risk estimate statistics cannot be computed. They are only computed for two groups of data

**Table 4:** Collinearity diagnostic for all indexes which included in multivariate Cox analysis in HBV-ACLF patients

Model	Collinearity statistics		Model	Collinearity statistics	
	Tolerance	VIF		Tolerance	VIF
Age	0.814	1.228		0.852	1.174
Male	0.833	1.200		0.863	1.159
Methylprednisolone	0.856	1.168		0.856	1.168
**MELD**	**0.048**	**20.674**		**–**	**–**
ALT	0.778	1.285		0.787	1.270
TBIL	0.324	3.088		0.772	1.295
Blood urea nitrogen	0.504	1.986		0.504	1.986
Creatinine	0.081	12.282		0.485	2.063
Triglyceride	0.795	1.258		0.796	1.257
Lymphocyte percentage	0.117	8.521		0.118	8.494
Monocyte percentage	0.336	2.979		0.336	2.974
Neutrophil percentage	0.103	9.672		0.104	9.644

**Fig. 3:**
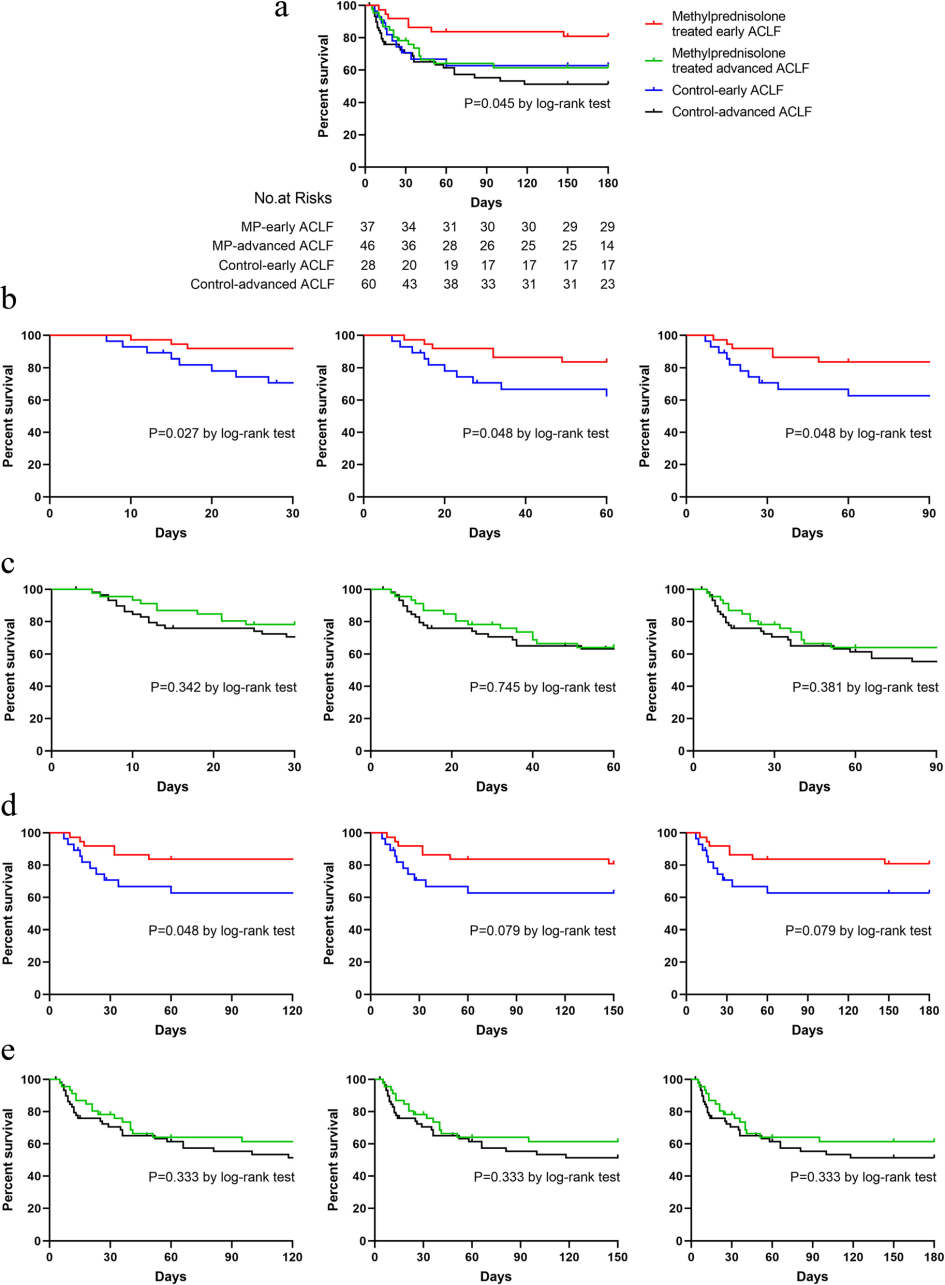
The efficacy of MP in early ACLF (those without ascites, without encephalopathy) and advanced stage ACLF. **a** The survival analysis of patients with ACLF in different four groups. **b** The survival analysis of patients with ACLF between methylprednisolone group and control group in early ACLF (those without ascites, without encephalopathy) during 30 days, 60 days, and 90 days. **c** The survival analysis of patients with ACLF between methylprednisolone group and control group in advanced ACLF during 30 days, 60 days, and 90 days. **d** The survival analysis of patients with ACLF between methylprednisolone group and control group in early ACLF (those without ascites, without encephalopathy) during 120 days, 150 days, and 180 days. **e** The survival analysis of patients with ACLF between methylprednisolone group and control group in advanced ACLF during 30 days, 60 days, and 90 days

The subset analysis for the efficacy of MP in those without ascites, without encephalopathy, is a useful way to show the efficacy of MP in early ACLF. Significant differences were observed (*P* = 0.045) in the survival rates among MP-treated early ACLF (81.1%), MP-treated advanced ACLF (63.0%), control-early ACLF (64.3%), and control-advanced ACLF groups (55%) at 6 months. The survival rate of MP- treated early ACLF group was higher than control-early ACLF on 30 days [HR (95% CI) 3.981 (1.192–13.300); *P* = 0.027], 60 days [HR (95% CI) 2.658 (0.971–7.276); *P* = 0.048], 90 days [HR (95% CI) 2.658 (0.971–7.276); *P* = 0.048], and 120 days [HR (95% CI) 2.658 (0.971–7.276); *P* = 0.048] (Fig. 3).

### Six-month secondary outcome analysis

#### Improved survival correlates with lymphocyte percentage, monocyte percentage, and LMR

**Fig. 4:**
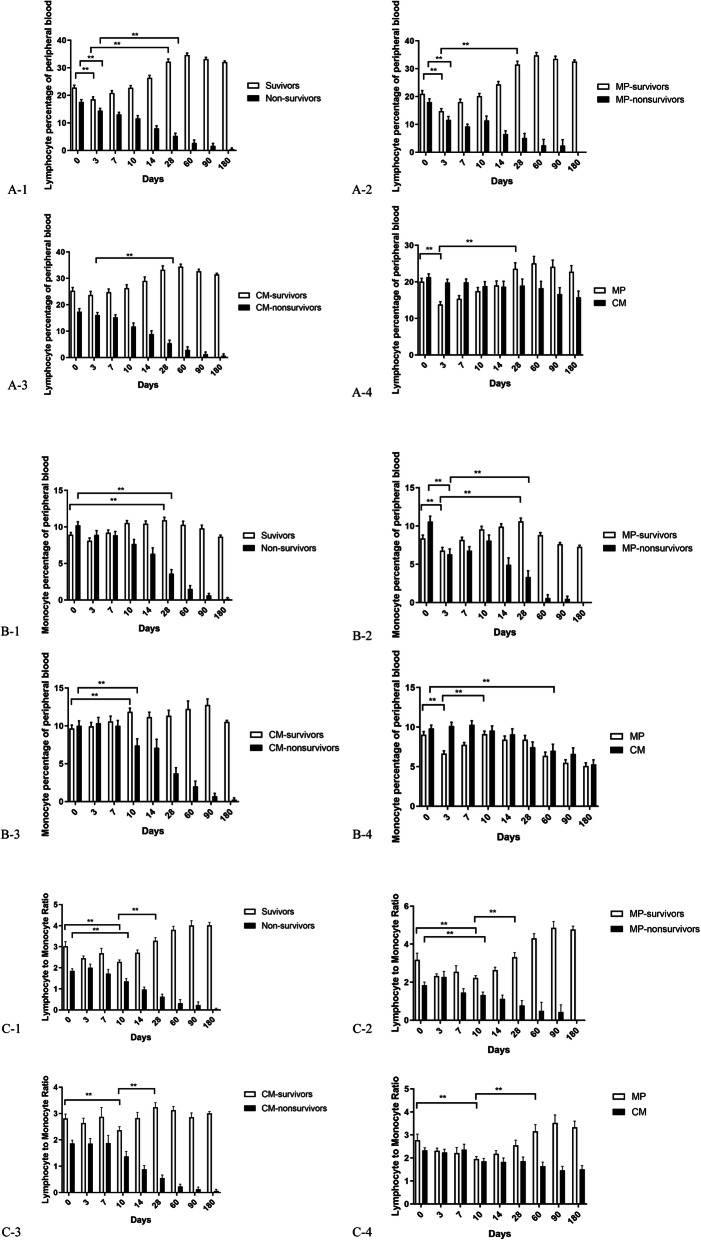
Influences of clinic indices on the outcome of HBV-ACLF. **A-1** Distribution of lymphocyte percentage between survivors and non-survivors in the 171 ACLF patients, **P < 0.01. **A-2** Distribution of lymphocyte percentage between survivors and non-survivors in the MP group, **P < 0.01. **A-3** Distribution of lymphocyte percentage between survivors and non-survivors in the control group, **P < 0.01. **A-4** Distribution of lymphocyte percentage between MP group and control group, **P < 0.01. **B-1** Distribution of monocyte percentage between survivors and non-survivors in the 171 ACLF patients, **P < 0.01. **B-2** Distribution of monocyte percentage between survivors and non-survivors in the MP group, **P < 0.01. **B-3** Distribution of monocyte percentage between survivors and non-survivors in the control group, **P < 0.01. **B-4** Distribution of monocyte percentage between MP group and control group, **P < 0.01. **C-1** Distribution of lymphocyte to monocyte ratio (LMR) between survivors and non-survivors in the 171 ACLF patients, **P < 0.01. **C-2** Distribution of lymphocyte to monocyte ratio (LMR) between survivors and non-survivors in the MP group, **P < 0.01. **C-3** Distribution of lymphocyte to monocyte ratio (LMR) between survivors and non-survivors in the control group, **P < 0.01. **C-4** Distribution of lymphocyte to monocyte ratio (LMR) between MP group and control group, **P < 0.01

As shown in Fig. 4, the survivors had a higher lymphocyte percentage and a lower monocyte percentage in peripheral blood compared to the non-survivors at baseline (*P* < 0.01). In the MP group, the survivors had a higher LMR in peripheral blood compared to the non-survivors at baseline (*P* < 0.01). The MP group exhibited a rapid decrease in lymphocyte percentage, monocyte percentage, and LMR in peripheral blood during treatment compared to control group. Subsequently, the survivors in the MP group displayed a continuous increase in the above indices compared to the non-survivors (*P* < 0.01). The restoration of immunity was characterized by the recovery of lymphocyte percentage, monocyte percentage, and LMR in peripheral blood after MP treatment.

**Fig. 5:**
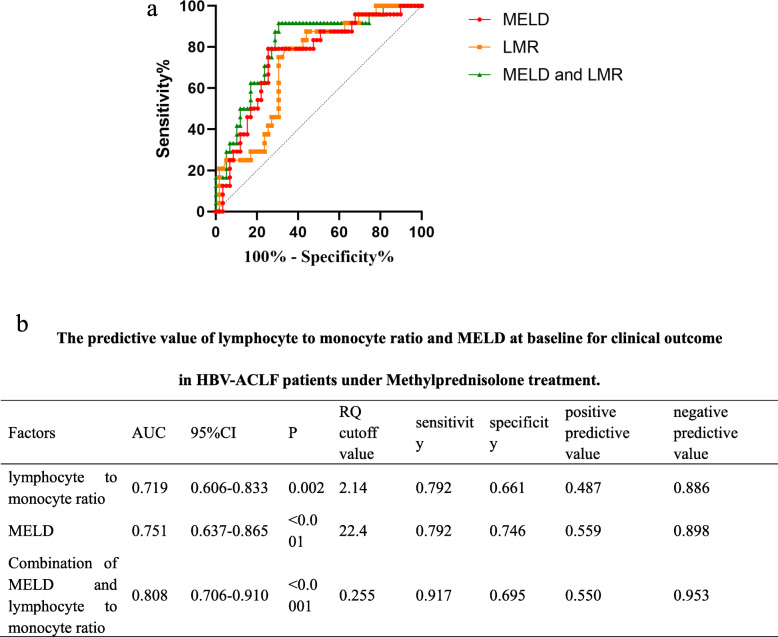
**a** Based on ROC, predictive value of LMR+MELD was higher than any single index for mortality of methylprednisolone treatment. **b** The predictive value of lymphocyte to monocyte ratio and MELD at baseline for clinical outcome in HBV-ACLF patients under methylprednisolone treatment

Based on ROC curve, MELD+LMR had a better predictive value for prognosis of HBV-ACLF under MP treatment. The RQ cutoff value was 2.14 for LMR, 22.4 for MELD, and 0.255 for MELD + LMR (Fig. 5).

#### Effect of MP on HBV DNA and liver function

**Fig. 6:**
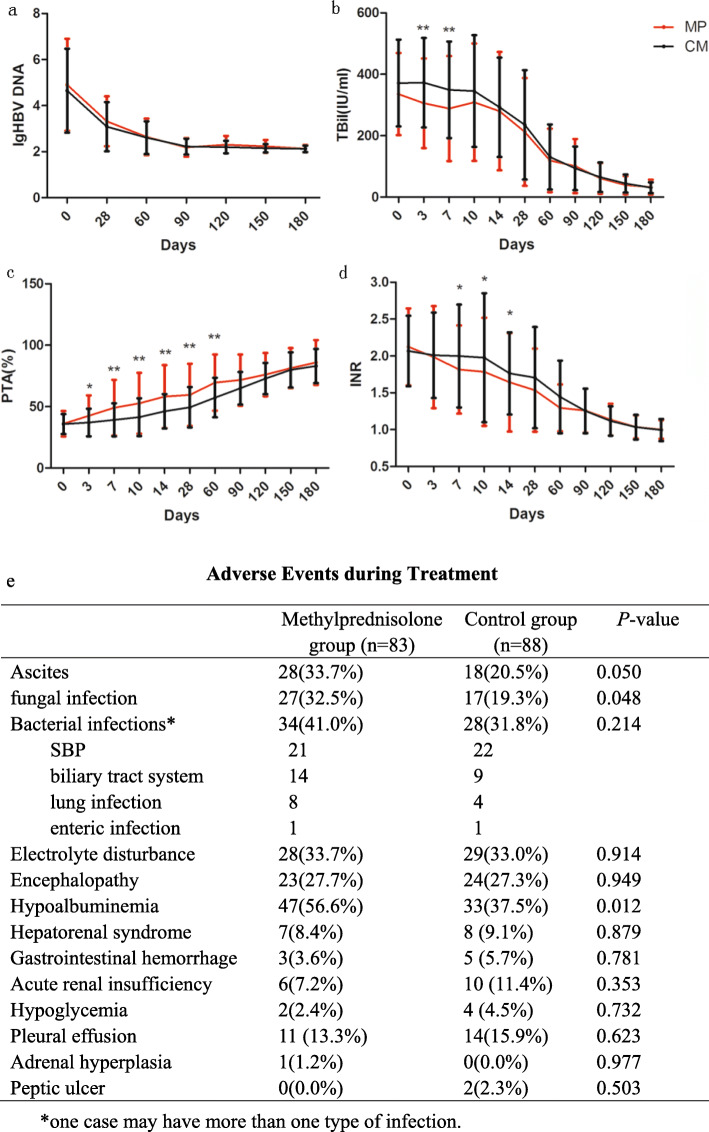
Effect of methylprednisolone on HBV-DNA and indicators of liver function. No significant difference in serum HBV DNA level was observed between the two groups (**a**). Serum bilirubin was significantly lower on days 3 and 7 (**b**); PTA was higher on days 3, 7, 10, 14, 28, and 60 (**c**); and INR was lower on days 7 and 10 (**d**) in methylprednisolone group than control group. Error bars: ± 1 SD, *P < 0.05; **P < 0.01. **e** Adverse events during treatment

No significant difference in HBV DNA quantity was observed between groups (*P* > 0.05, Fig. 6a). Compared to control group, serum bilirubin was lower on day 3 and day 7; PTA was higher on days 3, 7, 10, 14, 28, and 60; INR was lower on days 7, 10, and 14 in the MP group (Fig. 6b–d).

#### Adverse events

The incidence of hypoalbuminemia (56.6% vs. 37.5%, *P =* 0.012), fungal infection (32.5% vs. 19.3%, *P =* 0.048), or ascites (33.7% vs. 20.5%, *P* = 0.050) was higher in MP group compared with the control group. Forty-seven patients developed HE, 27.7% in the MP group whereas 27.3% in the control group (*P* = 0.949). The incidence of newly onset infection was 41.0% in the MP group whereas 31.8% in the control group (*P* = 0.214). Other adverse events including electrolyte disturbance, HRS, gastrointestinal hemorrhage, acute renal insufficiency, hypoglycemia, pleural effusion, adrenal hyperplasia, and peptic ulcer were comparable between groups (Fig. 6e).

## Discussion

ACLF is commonly accompanied by rapid progression, multiple organ failure, and low survival rate. Liver transplantation is the only treatment which has proven beneficial. However, the lack of donors and rapid disease progression limit its application [26, 27]. Therefore, there is an urgent need to find an effective and safe approach to ACLF. In this study, we found that MP improved the efficacy of standard treatment in HBV-ACLF, which could be a safe and effective treatment to HBV-ACLF.

The most common type of liver failure in the Asia-Pacific region is HBV-ACLF. The clinical stage of HBV-ACLF could be divided into four stages, early ascending stage, late ascending stage, platform stage, and recovery stage [28]. Immune injury is the main event in the early ascending stage. The pathogenesis in late ascending stage is related to immune injury, ischemia, and hypoxia injury. In the platform stage, body conditions reach an immunosuppression state.

Endotoxemia is a significant factor during the initiation of liver failure. Recent studies have reported that there existed an inflammatory cascade in the early stage of HBV-ACLF [29, 30]. The earlier systemic inflammatory response syndromes (SIRS) occur, the higher mortality would be. MP can stabilize hepatocyte membrane, suppress inflammation, and prevent further necrosis of hepatocyte [31]. Therefore, early application of MP therapy can suppress immune response. The inhibition of systemic inflammation improves the survival rate and delays rapid progression of patients with ACLF. Previously, we demonstrated MP improved the 28-day survival rate in HBV-ACLF [14]. Meanwhile, our previous evidence suggested strongly that the higher myeloid DC (mDC) numbers at baseline and the recovery of mDC number at the end of treatment may be a prognostic marker for favorable response to MP treatment in ACLF patients. The dosage MP used in previous studies is about 1 mg/kg/day and the duration is about 3 days to 10 days [32, 33]. However, those studies were small in sample size, uncontrolled, and heterogeneous in the treatment. In our multi-center, prospective randomized controlled clinical trial, sample size is much larger than others. Meanwhile, some retrospective studies have shown that the initial dose of MP (1 mg/kg/day) did not increase adverse events or mortality [34–36]. Therefore, in order to increase the decline of the mDCs counts and make sure the recovery of mDCs at the end of treatment, we further increased the dosage of MP treatment, with initial dosage of MP increasing to 1.5 mg/kg/day, so as to get a stronger immunosuppressive effect at the early stage and further improve the prognosis of patients. It was observed that MP significantly decreased 6-month mortality of HBV-ACLF compared to control group (32.4% vs. 42.5%).

Mortality attributed to infection was similar across groups. Most of the adverse events in this trial were liver related. However, MP increases the incidence of fungal infection, hypoalbuminemia, and ascites. This may be related to its activation of the renin-angiotensin system and suppression of the immune response [36].

It was reported that corticosteroids may enhance HBV replication [37]. However, in our study, none of the patients on short course of MP exhibited increased HBV-DNA, consistent with Zhang and Fujiwara’s studies [32, 33]. MP improves liver function, possibly by preventing endotoxin-induced secondary liver injury [38], inhibiting circulating toxic substances [39], and improving the functions of remaining hepatocytes [40].

The efficacy of MP treatment is also primarily associated with the timing of MP administration [28]. In this study, due to long duration for primary care in other hospital, the patients were not timely transferred to our centers; the median time of onset for ACLF was 16 days in the MP group whereas it was 20 days in the control group. Therefore, we failed to find the significance of onset time to guide early MP therapy. It is a limitation for our study. More studies should be proceeded to explore this issue in the future. Meanwhile, age and MELD score are not well balanced between the two groups (*P* = 0.121; *P* = 0.132) and it should be quoted as a limitation. Therefore, we further did propensity score matching analysis on age and MELD score to assess the baselines. It showed that Methylprednisolone group (83) could all fuzzy matched by control group (Supplementary Fig. 1).

## Conclusions

In conclusion, MP therapy is an effective and safe clinical strategy in HBV-ACLF, increasing the 6-month cumulative survival rate.

## Supplementary information

**Additional file 1: Fig. S1.** The propensity score matching analysis on age and MELD score to assess the baselines. It showed that Methylprednisolone group (83) could all fuzzy matched by control group.

## Data Availability

All data generated or analyzed during this study are included in this published article.

## Abbreviations

ADV: Adefovir
AE: Acute exacerbation
AFP: Alpha-fatal protein
ALT: Alanine aminotransferase
AST: Aspartate aminotransferase
CHB: Chronic hepatitis B
ETV: Entecavir
HBV: Hepatitis B virus
HBV-ACLF: HBV-related acute-on-chronic liver failure
HBeAg: Hepatitis B virus e antigen
HBsAg: Hepatitis B virus surface antigen
HE: Hepatic encephalopathy
HRS: Hepatorenal syndrome
INR: International normalized ratio
LAM: Lamivudine
LdT: Telbivudine
LMR: Lymphocyte to monocyte ratio
MELD: Model for end-stage liver disease
MP: Methylprednisolone
NAs: Nucleoside analogs
NH3: Blood ammonia
PT: Prothrombin time
PTA: Prothrombin activity
SBP: Spontaneous bacterial peritonitis
TDF: Tenofovir
