# Supplementary material for: The efficacy and safety of methylprednisolone in hepatitis B virus-related acute-on-chronic liver failure: a prospective multi-center clinical trial
Source: BMC Med. 2020 Dec 8;18:383. doi: 10.1186/s12916-020-01814-4 (PMC7722342; doi:10.1186/s12916-020-01814-4)
Supplement: Supplementary file 1 — Additional file 1: Fig. S1. The propensity score matching analysis on age and MELD score to assess the baselines. It showed that Methylprednisolone group (83) could all fuzzy matched by control group. [file 12916_2020_1814_MOESM1_ESM.docx]

**Supplementary Figure 1**

| **Case Control Matching Statistics** | |
| --- | --- |
| **Match Type** | **Count** |
| **Exact Matches** | **0** |
| **Fuzzy Matches** | **83** |
| **Unmatched Including Missing Keys** | **0** |
| **Unmatched with Valid Keys** | **0** |
| **Sampling** | **without replacement** |
| **Log file** | **none** |
| **Maximize Matching Performance** | **yes** |

| **Case Control Match Tolerances** | | | |
| --- | --- | --- | --- |
| **Match Variables** | **Value** | **Fuzzy Match Tries** | **Incremental Rejection Percentage** |
| **Exact (All Variables)** | **.** | **226.000** | **100.000** |
| **PS** | **.100** | **226.000** | **63.274** |
| **Tries is the number of match comparisons before drawing. Rejection percentage shows the match rejection rate. Rejections are attributed to the first variable in the BY list that causes rejection.** | | | |
